# Another market segment: sport event tourism by disabled athletes

**DOI:** 10.1186/s13102-024-00914-5

**Published:** 2024-05-28

**Authors:** Seokmin Yun, Kyungjin Kim, Sangyung Lee, Young Hoon Kim

**Affiliations:** 1 Department of Special Physical Education, Yeungnam University, Gyeongsan-Si, 38541 Korea; 2 Department of Adapted Physical Education, Korea National Sport University, Seoul, 05541 Korea; 3 National Institute of Green Technology, Seoul, 07328 Korea; 4 School of Business, College of Charleston, Charleston, SC 29424 USA

**Keywords:** Sports event tourism (SET), Disabled athletes (DA), Life satisfaction, Emotional happiness, Escapism, Perceived value and future intention

## Abstract

Sports and tourism are connected in various parts of economies, cultures, and nations. However, there has been a limited understanding of sports event tourism involving disabled athletes. This study explored the behavioral and socio-demographic implications of the disabled sports event tourism and investigated the motivational factors involved. An in-person survey was administered to 108 disabled athletes who attended the summer and winter Paralympics. A Structural Equation Model was used to determine the association between the factors of life satisfaction, emotional happiness, escapism, perceived value, and future intention, using the Monte Carlo parametric bootstrapping method to test significance of direct and indirect effects. Cronbach was acceptable because it exceeds 0.70 which satisfies the cut-off of confirmatory factor analysis. In addition, the individual values of average variance extracted (AVE), were greater than 0.50 (0.72) which meets the requirement and the convergent validity of all the constructs. The results showed that life satisfaction had a significant direct effect on future intentions. Emotional happiness had a significant direct effect on the perceived value of an event. Escapism had significant direct effects on perceived value and future intentions. Perceived value significantly influenced future intentions. The relationship between emotional happiness and future intention was fully mediated by perceived value. However, the relationship between escapism and future intention was only partially mediated by perceived value. The results of this study are valuable for developing future management and marketing policies for disabled athletes and tourists to advance the existing sports event tourism and disabled athlete’s behavior studies.

## Introduction

### Introduction

Sports Event Tourism (SET) is defined as traveling for the specific objective of participating in events or activities related to sports [1]. As indicated in previous studies [2, 3], sports tourism (here SET) has been formally or informally established as a subdiscipline of sports management. As Hudson [4] emphasized, sports event tourism has mostly a positive economic impact on destinations and has been highlighted as one of the most important tools to develop destinations [5]. Furthermore, sports event tourism can be a significant and decidedly sought-after market for destinations [6]. According to Kadam and Deshmukh [7], the global market size of sports (event) tourism was approximately $300 Billion in 2020 and is estimated to triple by 2030. Sports event tourism is interrelated with various parts of economies, cultures, and nations. Sports event can facilitate tourism and destination development by promoting the interests of spectators and stakeholders and contributing to the overall prominence of a destinations’ infrastructure and social exchange [1, 5]. According to Williams et al. [5], it is essential for sporting organizations to develop an actionable framework to stimulate near and longer-term strategic outcomes for destination hosting events. Kim et al. [1] also emphasizes that “A destination brand with strong equity leads to greater commitment in the form of loyalty and willingness to revisit that destination” (p. 1196). However, the studies on SET for destination are very limited in both qualitative and quantitative perspectives [1]. In particular, the studies on SET with and for disabled athletes are still in the infant stage [1, 5, 8, 9] while a few studies have attempted to understand non-disabled athlete’s behavior [1, 5, 10, 11].

The media representation of athletes with disabilities continues to be insufficient, even with just under 20% of the world population reporting a form of disability [12]. Blauwet and Willick [13] emphasized that the life scope and quality of disabled people has significantly improved for the last 30 years. Thus, it is highlighted that the influences of the media (e.g., sports event tourism) and communication practices are essential to raise awareness and challenge the traditional stereotypes of sports and disabilities [14–16]. Hua et al. [17] emphasized that some constraints (e.g., interpersonal, injury or cultural) can be overcome through the adoption of different strategies. For example, the participation of athletes with disabilities in the Paralympic Games has had a significant impact on their participation in sports. It has a lot to do with the injuries suffered by athletes with disabilities during Paralympic games. A study has shown that athletes with disabilities have high rates of injury during competition, which is not comparable to that of athletes without disabilities. Media will focus on research into the performance and injury resistance of athletes with disabilities [18]. This means developing methods to increase sports participation among people with disabilities and reduce or prevent injuries while improving performance. Additionally, perceptions of people with disabilities, such as the development of tourism for people with disabilities, may change. The media promotion of disabilities in sporting events (e.g., the Paralympic Games) may have a meaningful effect on social engagement and social sustainability; thus, emphasizing awareness and attracting more disabled athletes is very critical for destination and tourism development [8, 9]. People with disabilities’ exposure to media may improve the perception of disabled people among individuals without disabilities. Although there are some types of research on disabled tourism [19, 20], there has been a limited understanding of sports event tourism, especially by disabled athletes. To enhance the efficacy of sports tourism and destination, this study explores the behavioral and socio-demographic implications of sports events and investigates various related motivational factors of disabled athletes.

The objectives of this study are:

- To understand any challenges faced by the disabilities during their travel;
- To propose and examine the conceptual model by understanding the behavior of a disabled athlete at an event through the three-dimensional stages of human behavior: motivational factors, perceived value, and future intention;
- To validate the proposed model empirically, with collected data;
- To provide theoretical and practical suggestions to advance the understanding of disabled athletes’ behavior.

The sections that follow discuss the theoretical background of this study, the research protocol used to develop the measurement items and collection of data, the results that show the socio-demographic profiles of the participants, and if the model can be supported. Finally, theoretical, and practical suggestions will be provided for stakeholders, event managers, and athletes with disabilities.

### Theoretical background

#### Disabilities in tourism studies

Many previous tourism studies have emphasized that the disabled have the same or similar needs as people without disabilities and that this includes sports as well as travel [19, 21–25]. An increasing demand by the disabled and their families creates a substantial economic effect on tourism destinations from the growth of their travel spending as their social and financial levels have grown [22, 26]. Tourism becomes one of the most important parts of their life, including their family, with a better understanding and significant increase in social consensus about the disabled [27, 28]. It has been noted that tourism positively influences the lives of disabled individuals and the perceived value provided offers them the opportunity to interact with the non-disabled [29, 30]. It is estimated that over 1 billion individuals worldwide are disabled, or about 15% of the global population [31]. Thus, tourism and its related studies on the disabled are garnering greater interest from the tourism industry and academia. Hua et al. [17] suggested that worldwide, sports tourism is rapidly growing, with individuals with disabilities increasingly a class of tourist in need of attention. Disabled individuals engage in cultures that are primarily established for the able-bodied; thus, many disabled cannot freely engage in leisure activities. Society as a whole, whether disabled or not, requires the enjoyment that sports tourism provides and with varied choices the disabled demonstrate clear and well-defined goals for outdoor sports and leisure activities [17].

#### The application of the three-stages in consumer behavior model

The study of Consumer behavior is generally centered on how to engage consumers through targeted marketing promotional strategies, such as market segmentation, product differentiation, and distribution management. An understanding of consumer behavior provides a critical indicator of how people assess a given value (e.g., involvement and emotion) and where they should be positioned in consumer and tourism-related studies [32, 33]. It has been acknowledged that human behavior occurs as a process of emotional, physical, and elements which are determined through an individual’s sound and orderly manner of information gathering [34, 35].

**Fig. 1:**
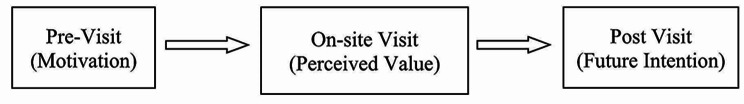
The proposed conceptual model

This study applies motivation, perceived value, and future intention to measure disabled athletes’ behavior while attending a sporting event. First, the motivation as a pre-visit process in each subject or environment (e.g., people, product, brand, surroundings) which can influence on-site evaluation (i.e., perceived value) [36, 37]. Second, the post-visit behavior (i.e., consumer’s future intention to revisit) can be associated with perceived value [38, 39]. From the review of three stages in consumer behavior, the study proposes of Motivation ? Perceived Value ? Future Intention framework to explore the association between disabled athlete’s experience components before, during, and after trips. In other words, this study proposes that disabled athlete’s behavior is positioned and measured by motivation (cognitive process), then value perception during the event (post-cognitive process), which subsequently influences their future intention (affective-conative process). The proposed model in Fig. 1 examines the relationship among the variables mentioned above.

#### Motivation

Social and psychological desires are connected to theories explaining the motives for attending sport events [40], with experiences creating positive feelings and emotional states, in addition to providing an awareness of fitting in to a group identity. This gratification can result from watching sporting events and is referred to as the degree spectators obtain entertainment, excitement, and enjoyment from observing a sporting event [40]. The following discusses the three motivation and behavior stages considered for this study:

##### Motivation for life satisfaction

Tourism experience is closely related to one’s general satisfaction with life [41]. Across different domains in life, tourism and leisure activity can improve individual happiness and quality of life [42–44]. Satisfaction is the degree of subjective utility or benefit obtained from the experience of tourism in relation to the economic, temporal, and social costs paid for the experience [45]. Overall, it is measured to examine whether a consumer has reached a state of satisfaction overtime after receiving a specific service [46]. Service satisfaction is derived from the customer’s individual perceptions and attributes of service expectations not resulting from the service itself [47]. Studies have identified positive associations with tourism satisfaction and after the purchase intentions [41]. For instance, Tzetzis et al. [48] noted tourism has a positive influence satisfaction on revisit intention. Yazıcı et al. [49] report that tourism satisfaction positively affects evaluation. Thus, motivation for (life) satisfaction can serve as an important predictor of future behavior, for example consumer intention to revisit. Extending these studies, this study posits disabled athletes can be motivated to have a relaxed, healthy, and pleasing lives by attending sports event tourism, and this life satisfaction motive can be significantly associated with perceived value and future intention. The research hypotheses are as follows:

H1a: Life satisfaction will significantly and positively influence perceived value.

H1b: Life satisfaction will significantly and positively influence revisit intention.

##### Motivation for emotional happiness

Happiness is a subjective experience that people feel in their life. It is an index measuring how satisfied we are with our life. Jeny and Varghese [50] described psychological happiness as a happy state without satisfaction, achievement, anxiety, and dissatisfaction. Euphoria is a personal tendency to experience positive emotions frequently and avoid experiencing negative emotions. It is an individual’s experience of pleasure, satisfaction, or a positive sense of well-being that we believe for meaningful life [51–54]. Abou-Zeid et al. [55] reported significant relationships among travel behavior and happiness, suggesting partaking in tourism undertakings can be an important driver of happiness, which in turn is central role to people’s physical and mental wellbeing. Elements of happiness include life satisfaction, subjective well-being, and quality of life [51, 56]. Young and Longman [57] defined happiness as satisfaction with the current living environment and conditions. It is said that happiness is deeply related to physical, mental, and social activities. Siegenthaler and Vaughan [58] stressed that leisure activity participants feel more pleasure and satisfaction than non-participants, and perceived higher quality of life through psychological well-being. Kim et al. [59] offered that tourist personal value and motivation can be efficient indicators of subjective happiness. Sports tourism for the disabled not only provides the disabled with opportunities to meet non-disabled people but also encourage them to compete with non-disabled athletes fairly and equally. Hence, studies have shown that positive emotions are generated through specific leisure experiences such as tourism to improve the overall value of life [60]. Thus, it has been confirmed that positive emotions are generated through specific leisure experiences such as tourism, which eventually improve the overall value of life [60] :

H2a: Emotional happiness will significantly and positively influence perceived value.

H2b: Emotional Happiness will significantly and positively influence future intention.

##### Motivation for escapism

Escape in sport tourism is a state of being completely engaged in the surrounding environment through active participation in experiential activities [30]. Escape is one of the major factors of motivation in the tourism studies [61]. According to Bello and Etzel [62], tourism is divided into an educational experience area and an area of familiarity and relaxation, and these experience areas include escape, novelty, and entertainment. Cohen [63] also claimed that experiential tourism was a component of escape from ordinary daily life and novelty to experience a new world. In addition, in the study of Oh et al. [64], the aesthetic factor had the highest relevance to the tourism experience, followed by escape. International travel has more and stronger hedonic motivation and satisfaction than domestic travel through escapism [65]. The deviant experience through tourism has a positive effect on perceived values [30], and these values are also used as a moderating variable for behavioral intention and various antecedent factors [54, 66, 67] :

H3a: Escapism will significantly and positively influence perceived value.

H3b: Escapism will significantly and positively influence future intention.

#### Perceived value

Value (or perceived value) has been defined variously in different concepts and structures. Zeithaml [68] defined it as a general assessment of whether a specific service is appropriate given what tourists perceive to be giving and receiving. In addition, Ezekiel [69] defined it as the difference of benefits a tourist obtains from using a specific service and the tangible and intangible cost paid for using it. The perceived value has been generally measured for monetary assessment, but now it should be assessed for various social concepts and structures. Measurement of a single value has been raised as lacking validity. Multidimensional values such as transaction, acquisition, cost of money, quality, emotional, social, and functional are being developed [70–74]. For instance, Lee et al. [75] developed a tourism program for the demilitarized zone (DMZ) region, a special zone between the two Koreas and divided the perceived value into three sub-values (i.e., overall, functional, and emotional) to measure the recommendations of tourist destinations. By studying the satisfaction of tourism, the result indicated that tourists were satisfied through functional and emotional value. Hwang and Hyun [76] further examined the positive relationship of sports tourism experience with perceived value. Extending the literature, this study proposes a relationship of sporting event tourism perceived value with other underlying factors such as life satisfaction, emotional happiness, and escapism:

H4: Perceived value will significantly and positively influence future intention and mediate relationships among the three motivational factors and Future Intention;

H4a: Perceived value will mediate satisfaction and future intention.

H4b: Perceived value will mediate emotional happiness and future intention.

H4c: Perceived value will mediate escapism and future intention.

#### Future intention

**Fig. 2:**
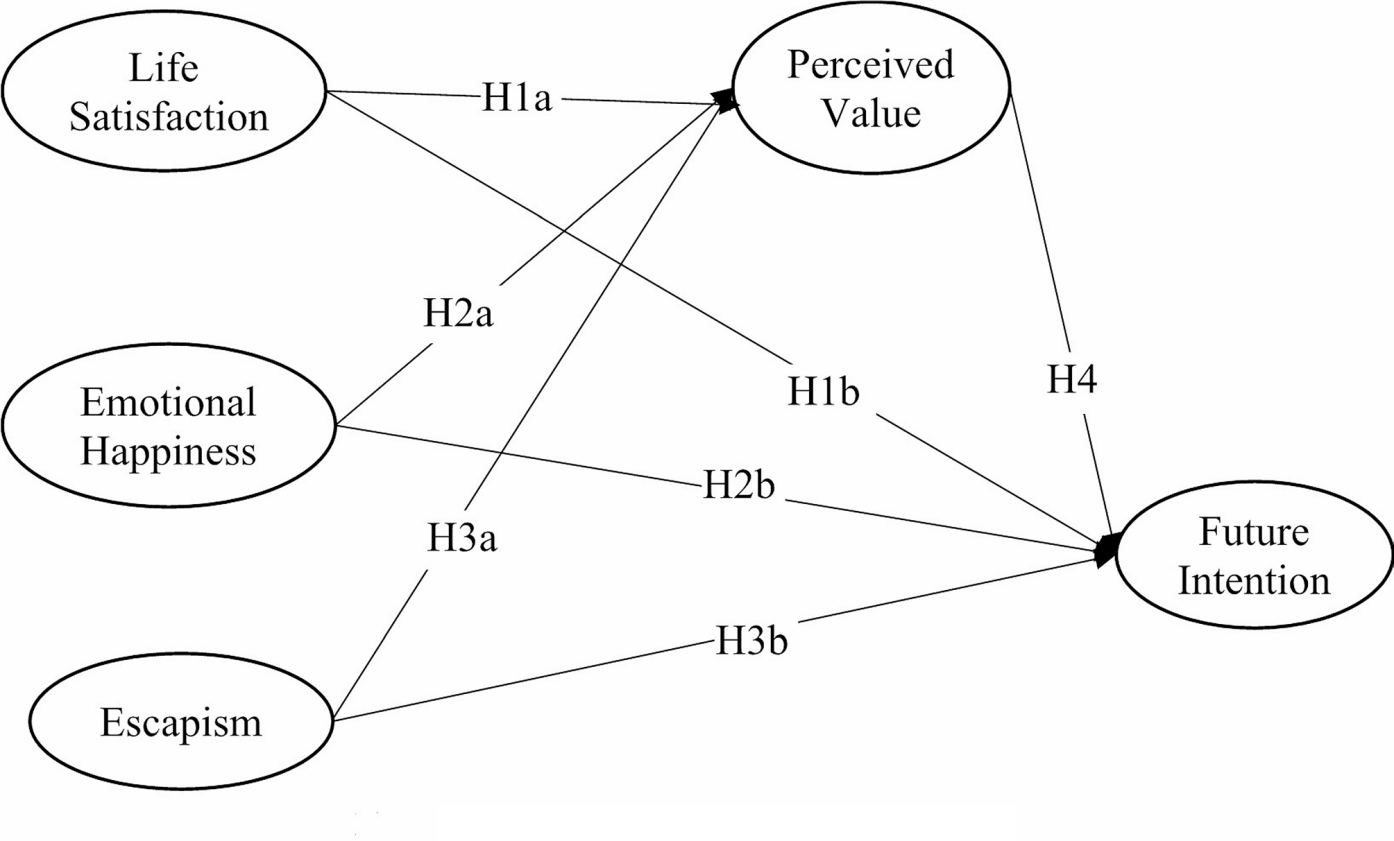
Conceptual framework with hypotheses

Studies on tourist’s future intention suggest individuals’ inclination to revisit and deliver positive word-of-mouth. Future intention can greatly influence the decision-making of visitors to visit the place of consumption or the area to be visited [77]. Many previous studies have investigated various factors that have significant impacts on future intentions, including affection, destination image, branding, perceived value, and satisfaction. For instance, Oshimi and Harada [78] identified the positive influence of affection towards the event, image of host city, and image fit on word-of-mouth of international sporting event. Saragi et al. [79] identified the role of destination branding and experiential marketing on tourist future intentions who visited Jakarta Province. Allameh et al. [80] validated the positive relationships between perceived quality and value, destination image, satisfaction, and future intention in the context of sports tourism. Extending these studies, the current research proposes the relationship between satisfaction, emotional happiness, and escapism towards positive future intentions (see Fig. 2).

## Methodology

### Survey and measurement item development

Both measurement and construction of survey questionnaire (i.e., items) are explained in Table 1. The survey was originally written in English and then, translated appropriately into Korea by English certified translating experts Again, all those items were refined and pre-tested by current research team. The reported reliability (i.e., internal consistency) and validation of the instrument have been acceptable and fit in acceptable ranges (See Table 2 for details). Socio-demographic questions, such as gender, age, income, were asked of the sample population (See Table 1). In addition, the study was approved by the Human Research Ethics Committee of the Korea National Sport University (IRB approval no. 20230921-089) and all participants signed an informed consent form.

**Table 1:** Construction of research question

Constructs	Item	Source
Life Satisfaction (4)	Participating in sport events is relaxed.	Armstrong, 2002; Park et al., 2016; Rode et al., 2018; Trail & James, 2001; Wann, 1995; Wild & Cant, 2015
	Participating in sport events is for a healthy leisure life.	
	Participating in sport events is for freedom.	
	Participating in sport events is for pleasure.	
Emotional Happiness (5)	Participating in sport event is always fun.	Armstrong, 2002; Park et al., 2016; Rode et al., 2018; Trail & James, 2001; Wann, 1995; Wild & Cant, 2015
	Participating in sport events allows you to have a happy time with your family.	
	Participating in sport events is for my happiness.	
	Participating in sport events is for psychological happiness.	
	Participating in sport events is for my life vitality.	
Escapism (4)	Participation in sport events provides opportunities for a sense of freedom.	Armstrong, 2002; Park et al., 2016; Rode et al., 2018; Trail & James, 2001; Wann, 1995; Wild & Cant, 2015
	Participating in sport events is to escape from everyday life.	
	Participation in sport events gives a sense of freedom.	
	Participation in sport events helps escape from daily life.	
Perceived Value (6)	Participating in sport events is superior to any other form of tourism.	Armstrong, 2002; Park et al., 2016; Rode et al., 2018; Trail & James, 2001; Wann, 1995; Wild & Cant, 2015
	Participating in sport events does not waste my time.	
	Participating in sport events gives me pleasure.	
	Participating in sport events has positively changed my life.	
	I perceive that participating in sport events has recharged my life.	
	I perceive that my life is enriched by participating in sport events.	
Future Intention (5)	I will participate in those sport events again that I am currently doing now.	Armstrong, 2002; Park et al., 2016; Rode et al., 2018; Trail & James, 2001; Wann, 1995; Wild & Cant, 2015
	I will participate in sport events that I am doing now with people around me.	
	If there is another sport events program, I will participate in it.	
	I will recommend the sport events program that I am currently participating into people around me.	
	I will tell other people around me about the sport events program I have participated in.	

### Data collection

Using an administered survey, we contacted a total of 241 disabled athletes from the South Korean national team for data collection. Among them, 142 provided usable data, and 108 questionnaires were retained for further analysis, representing a response rate of 76.06%. Participants were disabled athletes who participated in the 2020 Tokyo Summer Paralympics or the 2022 Beijing Winter Paralympics in sports such as table tennis, tennis, athletics, bowling, archery, basketball, paragliding, soccer, weightlifting, boccia, swimming, and badminton. The survey clearly explained the objectives and steps of the research and provided a paper-based questionnaire. Data were collected from June 2021 to May 2022. To ensure the rigor of data collection, subjects were recruited from various sources, and the sequence of questionnaire questions was randomly assigned. Participation was voluntary and anonymous. Participants were queried on several aspects, including life satisfaction, emotional happiness, escapism, value, and future intentions. Upon review, 108 questionnaires were deemed complete and usable for analysis.

### Data analysis

A Structural Equation Model (SEM) was used to examine the association between the factors of life satisfaction, emotional happiness, escapism, perceived value, and future intention, using the Monte Carlo parametric bootstrapping method (200 iterations) to test significance of direct and indirect effects. SEM is a statistical technique suitable for exploring comprehensive relationships between observed and latent variables within a conceptual model. SEM can minimize measurement errors while simultaneously analyzing the underlying mechanisms, enabling robust and reliable analysis of data consisting of multiple latent items. The analytical procedure necessary for SEM includes confirmatory factor analysis, which tests the reliability and validity of the statistics.

The number of respondents required for SEM depends on various factors such as the effect size, desired statistical power, complexity of the model, expected relationships among variables [81]. A commonly suggested guideline is sample-to-factor ratio of 15:1 or 20:1 [82], which means a minimum of 15 to 20 respondents must be considered for each independent factor in the model. Given that this study utilized five factors with 108 respondents, it is reasonable to conclude that the sample size aligns well with statistical guidelines.

## Results

### Demographics

Participant demographics are presented in Table 2. Respondents were primarily between 20 and 49 (81.6%) years of age, were more single (51.9%), and predominately male (71.3%). Eight 4% of respondents earned less than $3,000 monthly. More than a half of participants attended sports event tourism 1–2 times a year (60.2%). There were 3.7% of participants who had postgraduate education (See Table 2).

**Table 2:** Summary of demographics

Items	Frequency	Percent (%)
**Gender (***n* = 108**)**		
Male	77	71.3
Female	31	28.7
**Age (***n* = 108**)**		
20–29	26	24.1
30–39	33	30.6
40–49	29	26.9
50–59	19	17.6
Over 60	1	0.9
**What is your final educational level? (***n* = 108**)**		
High School graduate or less	51	47.2
University graduate or college attended	52	48.1
Postgraduate or higher	4	3.7
Others	1	0.9
**What is your job? (***n* = 108**)**		
Student	16	14.8
Office/Administrative position	2	1.9
Agriculture/Fishery	1	0.9
Production/Technical positions	2	1.9
Sales/Service	30	27.8
Specialized(professions)/Free positions	2	1.9
Self-employment/Management	54	50.0
Others	1	0.9
**Marital Status (***n* = 108**)**		
Single	56	51.9
Married	52	48.1
**Monthly Income (***n* = 108**)**		
Less than $2,000	39	36.1
$2,000–3,000	50	46.3
$3,000–4,000	14	13.0
$4,000–5,000	1	0.9
$5,000–6,000	3	2.8
Over $6,000	1	0.9
**How many children do you have? (***n* = 108**)**		
0	67	62.0
1	14	13.0
2	22	20.4
3 or more	5	4.6
**How many times a year are you involved in sports tourism? (***n* = 108**)**		
1–2	65	60.2
3–4	18	16.7
5–6	15	13.9
Over 7	10	9.3

### Confirmatory factor analysis

Reliabilities, factor loadings, and fit indices are shown in Table 3. According to Cronbach [83], reliabilities are considered acceptable if alpha exceeds 0.70 and for this study, they exceeded.70, which satisfies the cut-off of confirmatory factor analysis. When considering average variance extracted (AVE), the individual values were greater than 0.50, which meets the requirement and the convergent validity of all the constructs when using structural equation modelling. Factor loadings were greater than 0.40, satisfying discriminant validity. The model χ2 was 528.918 (*p* < .001) with a CMIN of 2.186, and other fit statistics such as CFI (0.910), NFI (0.848), IFI (0.911), RFI (0.827), and TLI (0.898) suggest at least a moderate fit (See Table 3).

**Table 3:** Factor loadings, reliabilities, and fit indices

Constructs	Factor loadings (Std estimates)	Cronbach alpha	Average variance extracted (AVE)	Composite reliability (CR)
Life Satisfaction	0.953	0.915	0.737	0.917
	0.953			
	0.826			
	0.671			
Emotional Happiness	0.911	0.921	0.720	0.927
	0.930			
	0.870			
	0.670			
	0.835			
Escapism	0.915	0.941	0.802	0.942
	0.839			
	0.922			
	0.904			
Perceived Value	0.871	0.966	0.828	0.967
	0.924			
	0.909			
	0.913			
	0.929			
	0.913			
Future Intention	0.855	0.930	0.729	0.931
	0.831			
	0.816			
	0.874			
	0.890			

### Path analysis

**Fig. 3:**
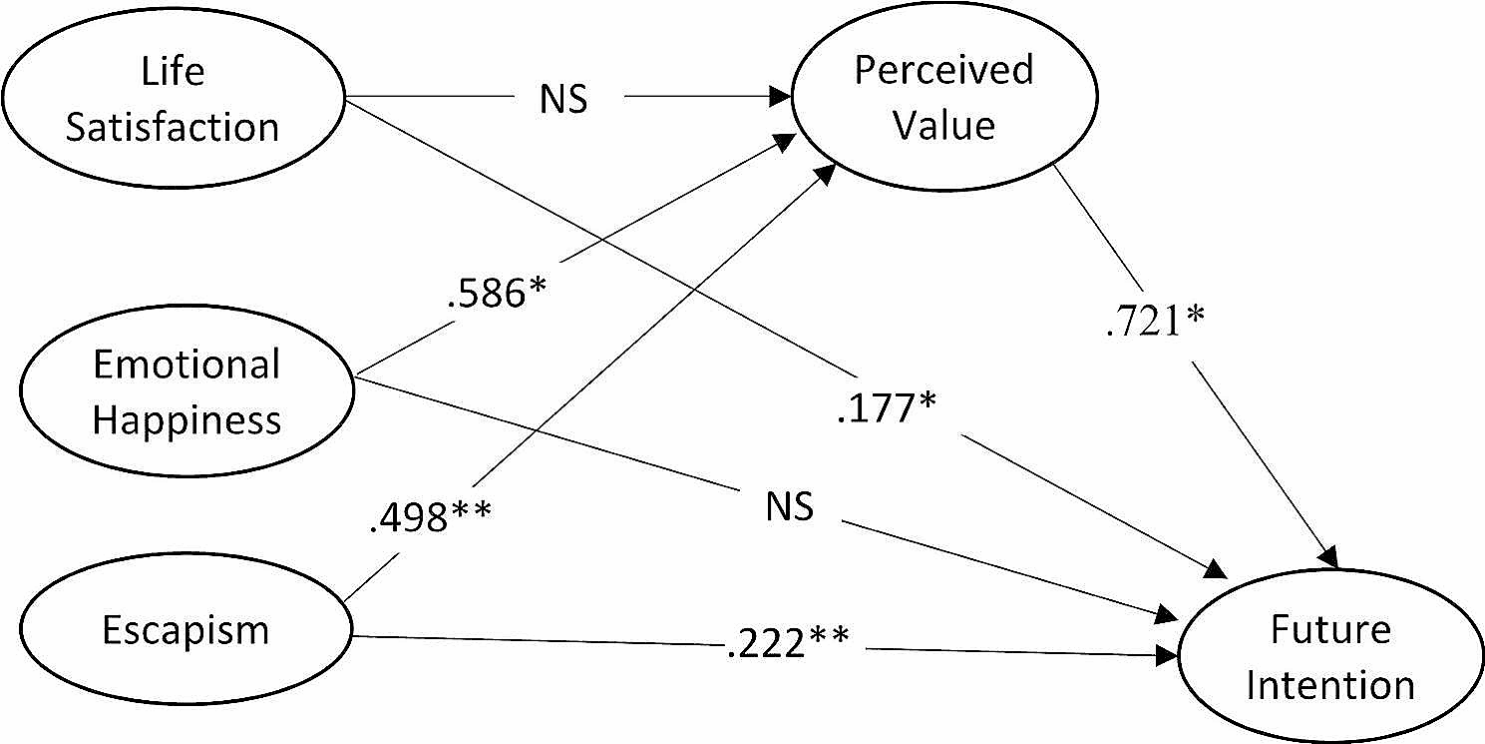
SEM overview (Direct effects)

Regression weights and direct, indirect, and total effects are shown in Tables, 4, 5, and Fig. 3, respectively. Life satisfaction had a significant direct effect on future intention (*p* < .05), while non-significant on perceived value. Emotional happiness had a significant direct effect on perceived value (*p* < .05), while non-significant on future intention. Escapism had significant direct effects on perceived value (*p* < .01) and future intention (*p* < .01). The direct effect of perceived value on future intention was significant (*p* < .05).

**Table 4:** Regression weights

Constructs	Dependent	Hypotheses	Unstandardized estimates	Standardized estimates	S.E.	Significance
Independent						
Life Satisfaction	Perceived Value	H1a	− 0.102	− 0.132	0.044	*
Emotional Happiness	Perceived Value	H2a	0.586	0.651	0.066	***
Escapism	Perceived Value	H3a	0.498	0.552	0.062	***
Life Satisfaction	Future Intention	H1b	0.177	0.258	0.034	***
Emotional Happiness	Future Intention	H2b	− 0.029	− 0.036	0.065	NS
Escapism	Future Intention	H3b	0.222	0.276	0.061	***
Perceived Value	Future Intention	H4	0.721	0.810	0.110	***

† * *p* < .05; *** *p* < .001, *NS* Non-Significant

### Mediation analysis

The mediation effects of each indirect path are shown in Table 5. First, the path between emotional happiness and future intention was significantly mediated by perceived value (*p* < .05). The total effect of emotional happiness to future intention became significant due to the presence of the mediator. In other words, the relationship between emotional happiness and future intention was fully mediated by perceived value. Second, the path between escapism and future intention was significantly mediated by perceived value (*p* < .01). A partial mediation was identified because both the direct (*p* < .01) and indirect effects (*p* < .01) were significant. Thus, perceived value accounted for a part of the relationship between escapism and future intention.

**Table 5:** Direct, indirect, and total effects

Independent	Dependent	Direct	Indirect	Total	Hypotheses
Life Satisfaction	Perceived Value	− 0.102		− 0.102	
	Future Intention	0.177*	− 0.074	0.104	H4a
Emotional Happiness	Perceived Value	0.586*		0.586*	
	Future Intention	− 0.029	0.423*	0.394*	H4b
Escapism	Perceived Value	0.498**		0.498**	
	Future Intention	0.222**	0.359**	0.581**	H4c
Perceived Value	Future Intention	0.721*		0.721*	

† * *p* < .05; ** *p* < .01

## Discussion

The conceptual model examines and tests the motivations of disabled athletes by using actual data from those who had attended international sport events or competitions (i.e., the 2020 Summer Tokyo and 2022 Beijing Winter Paralympics). The model, we believe, adds new knowledge by offering an innovative examination into the role of disabled athletes’ motivations, perceived value, and future intention how perceived value mediates these two measuring items. The findings of this study extended and confirmed the previous theoretical frameworks by emphasizing the critical research areas in life quality and satisfaction [42–44], emotional happiness [55, 60, 84], escapism [30, 61, 62, 65], perceived value [71–74, 76], and future intention [77, 79, 80, 85]. In particular, the methodology of this study extends tourism research beyond typical statistical measurement. With a significant growth of interest in tourism for disabled people, future research can focus on those subjects to understand and build unique behavioral models. This study proposed and applied the first conceptual model by understanding how disabled tourists are motivated in behavioral process in each stage.

The results of this study provide the following practical implications. From the perspective of SET, this study identified the influence of various pre-visit motivators of disabled athletic attendees on their on-site evaluation and post-visit intentions. The findings of this study suggest that the orientations of these attendees, such as their pre-visit motivations towards life satisfaction, emotional happiness, and escapism, can be closely related to their quality of life, time value, and health. Maybe, more detailed and life-based (e.g., health tourism) travel packages for the disabled athletes will have them motivated more while they are making decision. For example, value-driven approaches (e.g., free admission for disabled athletes if travel with more than three family members) could be another promotional strategies to increase their pre-visit motivation. In particular, hosting country can provide discounts on public transportation for returning visitors. It could be a promising strategy for boosting tourism and fostering positive relationships with revisit tourists. Again, it’s essential to carefully evaluate the potential benefits and drawbacks and consider the long-term sustainability of such initiatives and policies.

From a practical perspective, motivation is also associated with sport tourists’ higher persistence, emotions, the importance in participating, and gratification resulting from the sporting events; consequently, greater focus should be directed to profitable marketing strategies and practices that will influence sport tourists’ self-perceptions. Knowledgeable and determined sport tourists’ views on “getting in shape health benefits;”, “I can do it winning attitude”, “opportunities for better time management,” “traveling to places of interest,” and “chances to do something extraordinary” can be important elements for developing a successful sporting event promotional strategy through increased participants’ involvement [86, 87]. The systematic identification of SET’s impacts need further consideration because the mismatch between the target audience’s motives and the event’s concepts cannot produce a positive enhancement of on-site and post-visit outcomes. Any promotional strategies will enhance future intention of the disabled to attend the event. In addition, sports event organizers in the hosting country/nation should offer disabled athletes more compelling reasons to revisit the destination. For instance, the event organizers or management team could provide returning athletes and their families and friends with tailored touring packages, including opportunities such as locker room tours.

From the educational perspective, the outcomes of this study should lead us to understand how the disabled athletes are motivated to assess the value and plan (i.e., intention) for the next event. Thus, for the DAs, including the non-disabled, it is necessary to understand their behavior by measuring and analyzing their motivational factors. Many studies indicated that experiencing a new culture and learning new information is different in their behavior [88, 89] while any socio-psychological motives in tourism are similar for sport event travelers. In addition, more and additional factors should be studied and explored because sports event tourists experience and learn certain aspects of the foreign destination’s culture such as leisure activities, food, transportation, language, housing, arts, media, currency, and indigenous population [89, 90]. As previous studies have recognized the importance of motivation for understanding travel behaviors before (pre-visit), during (on-site-visit), and after (post-visit) site visits [90, 92], understanding of stakeholder’s motivations, including managers, workers, and residents are very pivotal [93]. In particular, sports event tourism with disabled athletes could be another research area in sport management as discussed earlier [2, 3, 94, 95].

From the marketing perspective, the disabled athlete’s values, including multidimensional values (transaction value, acquisition value, cost-money value, quality and value, emotional value, social value, functional value, etc.) need to be investigated and analyzed. Happiness and life satisfaction will be critical elements to promote disabled athletes and their family. According to Matson-Barkat et al. [96], the DAs would like to share or report their own emotional happiness which mutually empower them and reduce stigma related to disability. Promotional strategies using motivational factors (i.e., happiness and life satisfaction) will increase their intention to revisit in the future while happiness and pride, which eventually enhance self -confidence or actualization, were proved as critical emotions [96]. On the other hand, any marketing tools by inspiring and increasing affective positive feelings will attract an athlete’s tendency to attend sport events. Those marketing and promotional strategies can be maximized through social media. Hayduk and Walker [97] emphasized that it is easy to understand consumers’ demands, including instant reactions through social network communication channels.

## Limitations and future study

The results of this study cannot be generalized to a larger population because different ethnic and cultural groups could express varying motivations, values, and intentions. Future studies across countries may broaden the generalizability of tourist’s behavior. The depth of the questions, such as whether a participant has participated in any sporting events, may not have provided deep and rich information. Future studies should include an enhanced and more developed survey with more specific and detailed questions, such as more detailed questions assessing motivations or behavioral factors that lead to positive behavioral intentions.

In summary, the authors believe the proposed model and results can represent a first step in addressing the disabled athletes’ behavior research, but rather the futuristic approach to explore more from disabled tourists’ perspectives. Furthermore, the results from the proposed model may be useful in strategic planning for the sports events for the disabled (e.g., Paralympics) and its strategy for marketing and management.

## Conclusion

The study was designed to assess the disabled’s behavior while they are attending sporting events. The results of the study showed significant findings and particularly, the significant mediation effect of perceived value on future intention to visit. The overall results demonstrated that three motivations (i.e., life satisfaction, emotional happiness, and escapism), perceived value, and future intention have a strong and significant relationship. From a theoretical perspective, three stages of behavior (cognitive-affective-conative) were utilized to understand athlete’s behavior by integrating a theoretical framework. The proposed model was conceptually developed to understand consumer’s behavior (i.e., disabled athletes) by utilizing the cognitive-affective-conative stages and integrating theoretical framework. Interestingly, new term, “motivation for life satisfaction” was developed, modified, measured, and applied for the study. The item, “satisfaction” is generally measured for the affective stage. The finding of this study contributes to the existing sports event tourism and disabled athlete’s behavior studies in many ways.

## Data Availability

The data presented in this study are available on reasonable request from the corresponding author.
